# Accuracy and Feasibility of an Android-Based Digital Assessment Tool for Post Stroke Visual Disorders—The StrokeVision App

**DOI:** 10.3389/fneur.2018.00146

**Published:** 2018-03-28

**Authors:** Terence J. Quinn, Iain Livingstone, Alexander Weir, Robert Shaw, Andrew Breckenridge, Christine McAlpine, Claire M. Tarbert

**Affiliations:** ^1^Institute of Cardiovascular and Medical Sciences, University of Glasgow, Glasgow, United Kingdom; ^2^Glasgow Centre for Ophthalmic Clinical Research, Gartnavel General Hospital, Glasgow, United Kingdom; ^3^Medical Devices Unit, NHS Greater Glasgow and Clyde, Glasgow, United Kingdom; ^4^Glasgow Stroke Services, NHS Greater Glasgow and Clyde, Glasgow, United Kingdom

**Keywords:** apps, assessment, hemianopia, information technology, stroke, sensitivity, specificity, visual neglect

## Abstract

**Background:**

Visual impairment affects up to 70% of stroke survivors. We designed an app (StrokeVision) to facilitate screening for common post stroke visual issues (acuity, visual fields, and visual inattention). We sought to describe the test time, feasibility, acceptability, and accuracy of our app-based digital visual assessments against (a) current methods used for bedside screening and (b) gold standard measures.

**Methods:**

Patients were prospectively recruited from acute stroke settings. Index tests were app-based assessments of fields and inattention performed by a trained researcher. We compared against usual clinical screening practice of visual fields to confrontation, including inattention assessment (simultaneous stimuli). We also compared app to gold standard assessments of formal kinetic perimetry (Goldman or Octopus Visual Field Assessment); and pencil and paper-based tests of inattention (Albert’s, Star Cancelation, and Line Bisection). Results of inattention and field tests were adjudicated by a specialist Neuro-ophthalmologist. All assessors were masked to each other’s results. Participants and assessors graded acceptability using a bespoke scale that ranged from 0 (completely unacceptable) to 10 (perfect acceptability).

**Results:**

Of 48 stroke survivors recruited, the complete battery of index and reference tests for fields was successfully completed in 45. Similar acceptability scores were observed for app-based [assessor median score 10 (IQR: 9–10); patient 9 (IQR: 8–10)] and traditional bedside testing [assessor 10 (IQR: 9–10); patient 10 (IQR: 9–10)]. Median test time was longer for app-based testing [combined time to completion of all digital tests 420 s (IQR: 390–588)] when compared with conventional bedside testing [70 s, (IQR: 40–70)], but shorter than gold standard testing [1,260 s, (IQR: 1005–1,620)]. Compared with gold standard assessments, usual screening practice demonstrated 79% sensitivity and 82% specificity for detection of a stroke-related field defect. This compares with 79% sensitivity and 88% specificity for StrokeVision digital assessment.

**Conclusion:**

StrokeVision shows promise as a screening tool for visual complications in the acute phase of stroke. The app is at least as good as usual screening and offers other functionality that may make it attractive for use in acute stroke.

**Clinical Trial Registration:**

https://ClinicalTrials.gov/ct2/show/NCT02539381.

## Introduction

Visual impairment is reported to affect up to 66% of stroke survivors (1). Common visual deficits associated with stroke include visual field defects (hemianopia, quadrantanopia) (2), perceptual disorders (visual inattention/neglect) (3), and eye movement disorders (4). In a multicentre UK-based prospective cohort observation study of 915 patients, visual field loss was detected in 52.3% stroke survivors (2). A visual defect is a barrier to successful rehabilitation after a stroke and is associated with a poorer prognosis for rehabilitation (5, 6) as well as a diminished quality of life (2, 7). As stroke is predominantly a disease of older adults, those affected by stroke may also have co-existing eye problems, such as cataract and macular degeneration.

The importance of stroke-related visual problems has been recognized in guidelines from various professional societies. For example, the National Advisory Committee for Stroke (NACS) in Scotland, the body responsible for implementing a national strategy on stroke, have produced guidance for the screening and assessment of patients in the acute phase: The Best Practice Statement (BPS) for Vision Problems after Stroke (8). This paper was written in collaboration with experts (multidisciplinary medical practitioners and academics), voluntary organizations, and patient representatives, and is a comprehensive review of current pathways and best practice in the acute assessment of stroke associated with visual problems. Although there is no internationally agreed approach to vision assessment in stroke, the 2013 BPS recommended: screening assessment of visual fields with confrontation for all patients with stroke; use of two tests for visual inattention/neglect; and that results of these assessments should be explained to patients and caregivers. There is evidence that these recommendations are not universally adopted: Rowe et al. (9) in 2015 reported that only around 50% of health professionals looking after stroke patients used validated tests to screen for visual problems.

Advances in technology can provide solutions in situations where healthcare practice or delivery is not achieving desired standards. Mobile technology has become a ubiquitous part of modern life and has an increasing role in healthcare. Several studies have evaluated the physical properties of tablet devices (10, 11) with respect to visual assessment, evidencing a comparable performance with contemporary clinical standards (12).

We developed an app-based tool (herein referred to as StrokeVision) to try to meet the requirements of the BPS guidance, and improve stroke service performance in screening for visual impairment after a stroke (13). Our resource exploits the recent rapid advancements in mobile hardware and software to provide tools for assessment and education in stroke. The multimodality functioning has been developed by a multidisciplinary team of healthcare professionals, scientists, and engineers, and draws on the valuable input of patient and carer groups. In addition to the assessment function described in this paper, the app has education and patient information materials. A unique feature of the app is that data from visual assessments can be used to simulate the visual problems experienced by the user through use of the tablet video camera.

In developing StrokeVision, our aim was to directly address the recommendations of the BPS, creating a screening resource that (a) provides a rapid and accurate assessment of gross visual deficits in stroke survivors, (b) can be used at the bedside by non-specialist staff at the acute stage, (c) provides a means of integrating the results into the patient’s electronic record, (d) provides clear information to patients and carers about the identified visual defects and their implications, and (e) harnesses the features of touchscreen computer technology to aid testing in stroke patients with concurrent morbidities, such as poor vision, decreased fine motor control, and comprehension difficulties.

An important aspect of the development of an app for clinical use is validation in the intended user group. In this paper, we describe our validation study of our StrokeVision app with a particular emphasis on accuracy of the app for detecting visual field and inattention issues.

## Materials and Methods

### Aims

Our primary aim was to validate visual assessments in stroke using our StrokeVision digital platform, including visual field assessments and visual inattention/neglect assessments.

We designed a series of analyses around accuracy and feasibility of testing. Primary questions of interest were:

1. How does a tablet-based digital visual field test (StrokeVision app) compare against traditional clinical assessment of visual fields in terms of:
  - Accuracy of app against visual confrontation (the standard screening test in stroke practice).
  - Accuracy of app against formal kinetic perimetry (the gold standard assessment in stroke practice: Goldmann or Octopus Perimetry).
  - Feasibility/acceptability of assessments (test completion).
  - Time taken to complete the test.
2. How do tablet-based digital inattention tests (StrokeVision app) compare against traditional clinical assessment of inattention in terms of:
  - Accuracy of app against presentation of bilateral stimuli to confrontation (standard screen).
  - Accuracy of app against paper-based Line Bisection/Albert’s test (our chosen gold standards).
  - Feasibility/acceptability of assessments (test completion).
  - Time taken to complete the test.

### Study Design

We conducted a prospective test accuracy study, recruiting patients from various acute stroke sites across a University Hospital stroke service. The protocol was registered and is available at clinical trials (NCT02539381). We followed Standard for Reporting Diagnostic Accuracy (STARD) best practice in design, conduct, and reporting of our study. Our project was approved by West of Scotland NHS Research Ethics (15/WS/0071) and local Research and Development. All subjects gave written informed consent in accordance with the Declaration of Helsinki. The work was supported by the NACS. The funder played no part in data collection, analysis, or interpretation.

All test data were recorded on specifically designed and piloted case report forms. Participants were assigned a unique identifier. For each participant, testing was performed on the same day.

Visual field testing was performed in a dedicated clinical area with availability of perimetry equipment (Goldmann or Octopus). In contrast to white on white light projection into a Ganzfeld dome, as employed in conventional kinetic perimetry, the digital targets emerging from the periphery were black on white. The size III (4 mm^2^) Goldmann stimulus was tested without corrective lenses and standard lighting was used. We used standard operating procedures for transfer of patients from wards to the visual testing area and for testing. As many of the included patients had sustained, a recent stroke safety was a key concern. If the participant became fatigued or uncomfortable, testing was halted.

### Participants

We recruited participants from two sites within NHS Greater Glasgow and Clyde, where visual assessment of stroke was routinely performed. Recruiting sites included adult stroke units and outpatient clinics. Recruitment ran from 23/7/2015 to 28/6/2016.

The principal criterion for eligibility was clinical. We considered consecutive patients, where the attending clinical team requested formal assessment of vision, as we felt this was closest to how the app may be used in practice. Inclusion criteria were: clinical team felt suitable for assessment and requested assessment of vision; patient able to provide informed consent. We excluded patients with no spoken English.

A senior clinician recorded basic clinical and demographic details of participants: age, sex, acuity, pathological stroke classification (ischemic or hemorrhagic), and time since stroke.

### Index Test

**Figure 1:**
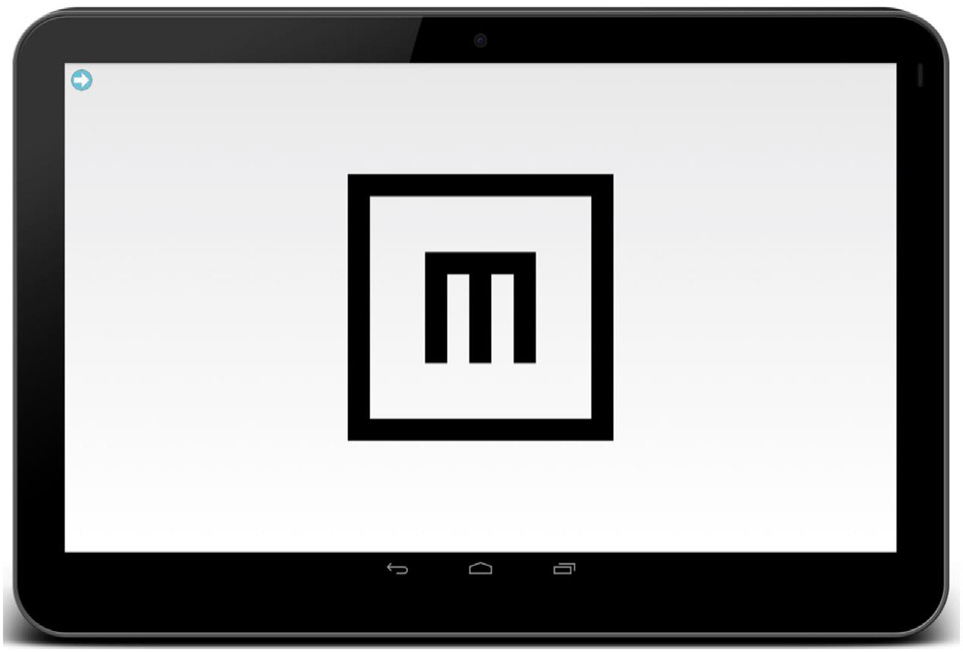
Tumbling E Near Acuity Test, adapted from the android-based distance optotype test, “peek acuity lite” (14.5). The patient is asked to swipe in the direction of the limbs of the E and a staircasing algorithm detects the threshold acuity level.

**Figure 2:**
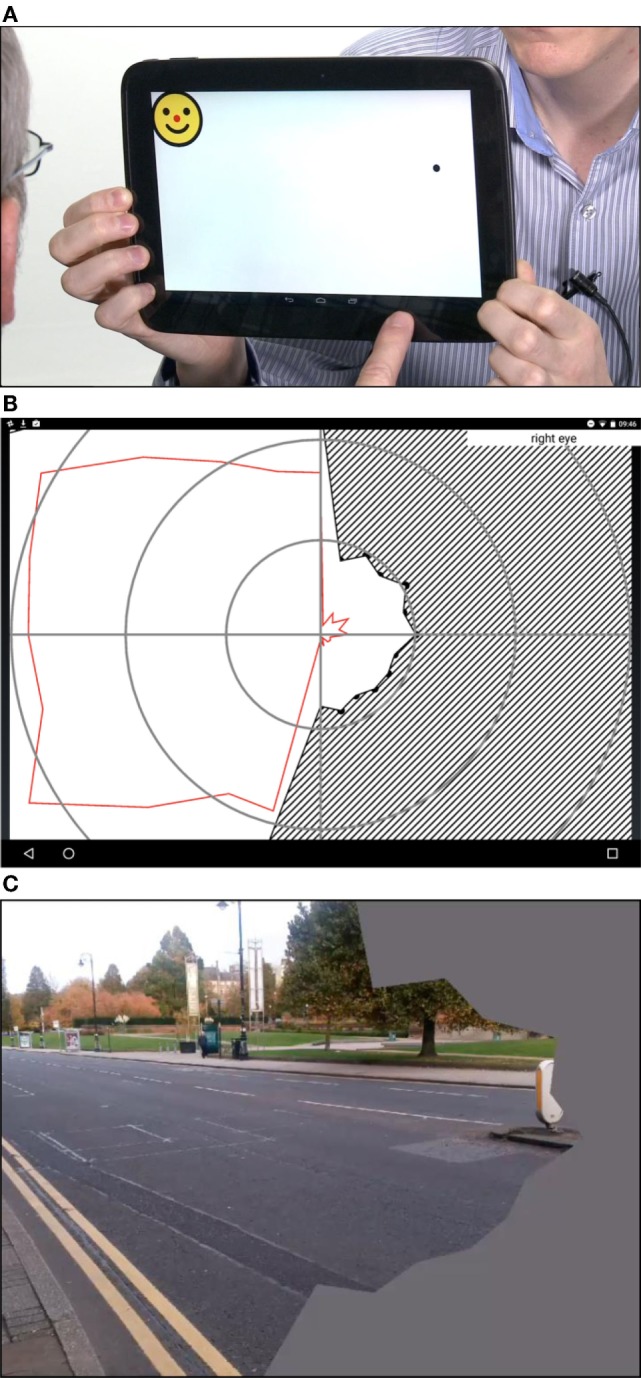
**(A)** The StrokeApp Visual Field test. The fixation target is the red nose of the smiling face graphic. The fixation target moves to the extreme corners of the device screen and the black circular target emerges from the periphery toward fixation. The peripheral target (black circle) subtends a similar angle to the Goldmann III target setting. The patient is encouraged to tap anywhere on the screen at the moment they detect the emerging peripheral target. The tester observes patient fixation, and if a fixation loss is detected, the screen is swiped to delete the previous input and re-test that point or quadrant. **(B)** The diagonal hashed lines indicate field defect once reaction time has been accounted for. The red line indicates limit of the visual field before reaction time has been accounted for. **(C)** StrokeSim. The detected perimetry plot is transposed as a digital filter to the feed from the device back-facing camera. The field within the defect is averaged to a single color.

**Figure 3:**
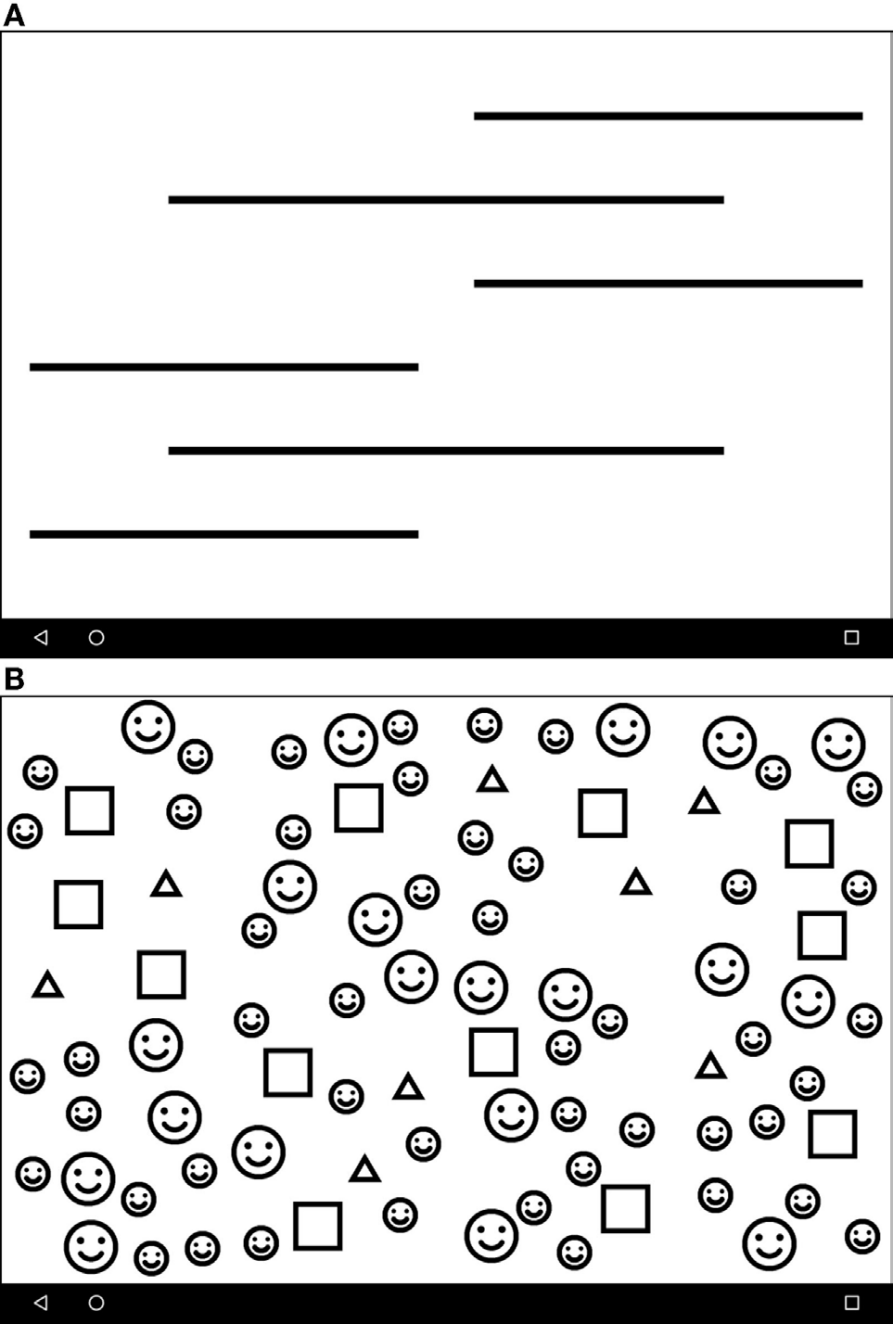
**(A)** The Line Crossing test screen. Line thickness is calibrated against the acuity score to allow testing in low-vision patients. The patient is asked to press the perceived center of each line. **(B)** The Face Cancelation Test. A second test of inattention, whereby the patient is asked to press the small faces. As previous, line thickness is calibrated to acuity score.

Our index test was the battery of vision tests within the StrokeVision app. The app was used with a Google Nexus 10 tablet device (Google Inc., Mountain View, CA, USA). The vision tests within the app comprised:

• An assessment of visual acuity (digital Tumbling E visual acuity assessment at 33 cm, Figure 1). This test was an altered version of “peek acuity” prototype build, with the letter sizing re-set to a 33 cm working distance (in contrast to the cited validation which investigated a distance version of the application) (14).
• Visual field test results for right and left eyes (Figures 2A,B).
• Digital tests of visual inattention: line bisection (Figure 3A) and face cancelation (Figure 3B).

The app-based tests were performed by fully trained research assistants masked to other clinical and test data. Based on the field testing, the app produces a visual field map that has a similar format to the output from formal perimetry (Figure 2B). Based on the inattention tests, the app produces a screenshot of the completed task (Figure 2C). These raw data were shared with a senior neuro-ophthalmologist (GW), masked to other test data for final classification.

### Reference Standards

As the intention of the app is to complement initial screening assessment, rather than replace diagnostic assessment, our primary analysis compared the app to traditional clinical assessment of visual field assessment using traditional confrontation, comprising a kinetic boundary test to moving fingers, where the patient reports when the examiner’s finger became visible when moved from outside peripheral vision of each quadrant toward fixation (from “unseen to seen”), comparing against the assessors own visual field.

Our screening test for visual inattention was also chosen to mirror routine clinical practice in the UK. We used bilateral visual stimuli (fingers moving) as the primary screen. Following assessment, the tester classified potential field deficit and recorded presence or absence of inattention, as is typical in clinical practice. These assessments were performed by a fully trained clinician (Stroke Unit doctor at Registrar or Consultant level, or an Orthoptist with specialist stroke training) masked to all other clinical and test data. For those patients recruited from the orthoptist outpatient clinic, “both eyes open” confrontation was not part of usual assessment and so was not routinely recorded. This information was recorded in a standard data inclusion sheet, designed to reflect standard methods of recording in everyday practice.

To further describe the accuracy of the app, we also assessed against accepted “gold standards.” Our gold standard for field assessment was formal perimetry using Goldmann (Haag Streit, Berlin) or Octopus (Interzeag Octopus 500 EZ, Haag Streit, Berlin) visual field testing apparatus. Choice of assessment device was based on availability at the time of testing.

We recognize that many potential tests of visual inattention are available. We selected those tests that are used in specialist stroke vision clinics within our service and that are recommended in guidelines (8). Our gold standard assessment for visual inattention was a series of pencil and paper tests, including Albert’s test (15), and an object bisection (line bisection) test featuring six horizontal lines offset from the center drawn in a word document. These tests are used for formal assessment within our stroke service. All gold standard assessments were either performed by a senior orthoptist specializing in stroke, or a fully trained senior vision scientist (CT). Assessors had no access to medical notes detailing visual diagnoses. Where possible, assessors were masked to results of both app and screening assessments. In a small number of cases where assessors were unmasked, the data was not excluded. The raw data from the perimetry and inattention assessments were shared with a senior neuro-ophthalmologist (GW), masked to other test data for final classification.

### Feasibility

To assess feasibility and acceptability of testing, we recorded the following metrics: time to complete each test; number of participants fully completing each test; and assessor perceived acceptability including patient compliance with test (recorded by the operator using a simple 0–10 scale, where 0 = completely unaccepatble and 10 = perfect acceptability). After all three assessment methods were performed, participants were asked to quantify acceptability of each assessment using a simple 0–10 scale, where 0 = completely unacceptable and 10 = perfect acceptability. Classification of assessment results: for the traditional testing (field and inattention performed at confrontation), classification was made at the point of testing by the examining clinician, and recorded on the data inclusion sheet, with the option of sketching any gross defect onto a four quadrant *pro forma* for each eye.

For the field maps produced by the app and formal perimetry, at the end of the data collection, order was randomized, and paired field test results (plots for right and left eyes) for (a) StrokeVision and (b) gold standard (Octopus or Goldmann) were presented to a Consultant Neuro-ophthalmologist (GW), who classified each pair as: (i) consistent with stroke-related defect; (ii) within normal limits; and (iii) non-gradable.

A free text field was included to allow further comment on the data arising from the app. Where a field plot was classified as “consistent with stroke-related defect,” the defect was sub-classified according to predefined criteria: homonymous defect: hemianopia/quadrantanopia/macula sparing, etc.

For the inattention data, paper-based gold standard and digital inattention results were similarly randomized and presented to the Consultant Neuro-ophthalmologist (GW). For the digital assessments, these were presented as a screenshot of the completed test generated by StrokeVision. These results were again graded as: (i) consistent with inattention (sub-classified into right or left-sided); (ii) within normal limits; and (iii) non-gradable.

### Analyses

Our analysis was pilot in nature, however, to ensure we had sufficient data for validity testing, we performed an illustrative power calculation to inform our proposed sample size. Based on sensitivity of confrontation for detection of a perimetry deficit of 0.5, and assuming the app will have a sensitivity of 0.7 (a value often taken as the minimum required for clinical utility) then at conventional alpha: 0.05 and beta: 0.2 we required a total of 50 participants for paired comparisons. Sample size was calculated using the online StatsToDo program (Chinese University of Hong-Kong, 2014 www.statstodo.com).

We used simple descriptive statistics to characterize the participants. For feasibility data we calculated: the proportion of patients able to complete each assessment; median time of each assessment modality and median acceptability scores for assessor and patient. We assessed agreement between the three assessment approaches (usual screening practice, gold standard assessments, and StrokeVision) using standard metrics to describe “accuracy” (sensitivity; specificity; and predictive values). We created a series of contingency tables of frequencies for visual field and visual inattention assessments against app and against each other. To allow further assessment of feasibility, we expanded the traditional “two by two” contingency tables with an intention to diagnose-based analyses that employed a third category of “unable to assess” for both index and reference tests, giving “3 × 3” tables. Analyses were conducted by two assessors masked to other clinical details and to each other’s scores. Results were compared and final results were consensus.

## Results

### Participants

We recruited 48 participants. Median age was 63 years (IQR: 54–72); *n* = 20 (42%) female; median visual acuity 0.2 logMAR (IQR: 0.1–0.4); median time from stroke 38 days (IQR: 7–99 days); *n* = 38 (80%) were ischemic infarcts and *n* = 15 (31%) of lesions were posterior cerebral territory. Of this group, 45 provided data that were used for accuracy based analyses (Table 1). According to gold standard assessment, 19 of 48 patients (39.6%) had field deficits and 3 (6.3%) had signs of visuospatial neglect.

**Table 1:** Test accuracy of Stroke Vision app compared to usual screening assessments for visual field deficits and visual inattention.

Comparison (A versus B)		Total “useable” data	Sensitivity (95% CI)	Specificity (95% CI)	PPV (95%CI)	NPV (95% CI)
					
A	B					
App	Visual fields confrontation	45	0.71 (0.48–0.89)	0.83 (0.64–0.95)	0.79 (0.54–0.94)	0.77 (0.56–0.91)
App	Bilateral stimuli confrontation	28	0.67 (0.09–0.99)	0.88 (0.69–0.97)	0.40 (0.05–0.85)	0.96 (0.78–1.00)
App	Formal perimetry	43	0.79 (0.54–0.94)	0.88 (0.68–0.97)	0.83 (0.59–0.97)	0.84 (0.64–0.95)
App	Formal inattention	44	0.67 (0.09–0.99)	0.98 (0.87–1.00)	0.98 (0.87–1.00)	0.67 (0.09–0.99)
Visual fields to confrontation	Formal perimetry	42	0.79 (0.49–0.95)	0.82 (0.63–0.93)	0.69 (0.41–0.84)	0.88 (0.69–0.98)
Bilateral stimuli	Formal inattention	26	0.50 (0.01–0.99)	0.87 (0.68–0.97)	0.25 (0.01–0.81)	0.95 (0.77–1.00)

*App, StrokeVision app; CI, confidence interval; PPV, positive predictive value; NPV, negative predictive value*.

*Total useable data, where paired app and reference standard test data were available for a patient*.

### Test Accuracy

Comparing the app to standard visual screening and gold standards gave accuracy measures that would be in the range usually considered moderate to good for a clinical test and that were comparable to the performance of usual screening test against gold standard (Tables 1 and 2). Participants struggled to complete all the test battery; the lower number of patients contributing accuracy data on bilateral stimuli reflects that this assessment was not standard in those recruited from orthoptics outpatient clinics (Tables 1 and 2).

**Table 2:** Contingency table describing test positive and negative results for StrokeVision app versus standard confrontation (visual fields), third row and column describes where participants were untestable or did not complete testing.

	Field deficit on app	No field deficit on app	No useable field data from app
Field deficit on confrontation	15	4	1
No field deficit on confrontation	6	20	1
No useable data from confrontation	2	0	0

### Feasibility/Acceptability

Median time to complete app-based field assessments was 300 s (IQR: 300–468, range: 180–600); this was the longest component of the app test battery. Total test time for app was 420 s (IQR: 390–588); compared with total test time of 70 s (IQR: 40–70) for screening tests and total test time 1,260 s (IQR: 1005–1620) for gold standard testing. Median assessor acceptability score for app-based field assessment was 10 (IQR: 8–10, range: 5–10), median patient acceptability score was 9 (IQR: 8–10, range: 5–10). Other metrics around acceptability and feasibility are presented in Table 3.

**Table 3:** Feasibility and acceptability of the Stroke Vision app.

	Median time to completion (seconds)	Median acceptability score (assessor)	Median acceptability score (patient)
App fields assessments	300 (IQR: 300–468, range: 180–600)	10 (IQR: 8–10, range: 5–10)	9 (IQR: 8–10, range: 5–10)
App shape cancelation	60 (IQR: 60–60, range: 30–300)	10 (IQR: 10–10, range: 7–10)	9 (IQR: 8–10, range: 6–10)
App line bisection	60 (IQR: 30–60, range: 20–300)	10 (IQR: 10–10, range: 7–10)	10 (IQR: 8–10, range: 3–10)
Formal perimetry	1,200 (IQR: 955–1500, range: 30–1,500)	8 (IQR: 5–10, range: 4–10)	7 (IQR: 5–10, range: 2–10)
Line bissection	30 (IQR: 25–60, range: 20–300)	8 (IQR: 10–10, range: 3–10)	10 (IQR: 8–10, range: 3–10)
Shape cancelation	30 (IQR: 20–60, range: 10–300)	10 (IQR: 9–10, range: 3–10)	9 (IQR: 8–10, range: 5–10)
Confrontation inattention	60 (IQR: 30–60, range: 20–300)	10 (IQR: 8–10, range: 5–10)	10 (IQR: 8–10, range: 4–10)
Screening inattention	10 (IQR: 10–10, range: 10–20)	10 (IQR: 10–10, range: 5–10)	10 (IQR: 9–10, range: 5–10)

### Adverse Events

No serious adverse events were reported. One participant developed nausea during perimetry and further testing was abandoned, one patient fell en route to testing area and this participant’s data were not included in final analyses. Many participants could not complete the full battery of screening, app, and gold standard assessments (see Tables 1 and 2). Reasons for test non-completion included difficulty in understanding instructions, difficulty in holding pen (for paper-based inattention tests), unable to position participant within the perimetry apparatus, fatigue.

## Discussion

Significant variation exists in visual screening practices and provision for stroke-related visual problems (9). We assessed a novel tablet-based app designed to harmonize screening practices and reflect best practice. We compared the app to both standard clinical practice on the stroke ward and also the gold standard assessment in a tertiary referral neuro-ophthalmology center. Our test accuracy data suggest that the app performs reasonably well against both standard bedside screening tests and formal gold standard assessments. Our data around feasibility and acceptability suggest that patients with stroke were able to complete app-based assessments and found this modality of testing to be acceptable.

The pattern of test accuracy data suggest that the app offers greater specificity than sensitivity. There is always a trade-off between sensitivity and specificity of a test, with no “optimal” value for these metrics, rather an evaluation of test properties should be made in light of the intended use of the test and the implications of a false positive and false negative results. For a screening tool, it may be more important to ensure that all cases are diagnosed and so higher sensitivity may be preferred. The sensitivity of both StrokeVision and confrontation in our data is higher than has been previously described for bedside “kinetic to finger” confrontation sensitivity, which has been reported at 40% (95% CI 30–51) when performed by an ophthalmologist and compared with full threshold automated static perimetry (16). It should be noted, however, that our study had significant methodological differences, with only stroke survivors being tested in our cohort (compared to a heterogeneous group of 138 eye clinic outpatients), and our gold standard being *kinetic* perimetry. While entrenched in clinical practice, and a basic competence trained and tested in medical school, confrontation field testing is performed in various ways, with significant technique-dependent variation in sensitivity reported. Our decision to use kinetic to finger confrontation is based on our impression of typical practice, though there is evidence that alternative techniques have increased sensitivity (16).

There are many approaches to the assessment of inattention, these include pencil and paper and electronic-based paradigms. There is no accepted, consensus on the preferred clinical assessment for visual inattention. We chose those tests used in our clinical service as comparators, namely bilateral simultaneous confrontation as screening test and two paper-based assessments (Albert’s and line bisection) as gold standard. With this regards, we have an “imperfect” gold standard as both of the paper-based assessments which can produce false positives and false negatives compared to multidisciplinary clinical assessment of functional inattention.

Our aim was to describe accuracy against both usual screening practice and gold standard assessment. When interpreting the data on accuracy against kinetic finger confrontation and bilateral simultaneous confrontation it must be remembered that these tests are themselves imperfect. Where there was disagreement between the app and the screening test, it is possible that the app was the true reflection of the patient’s visual status. It is reassuring that when tested against a gold standard the app was at least as good (and possibly improved upon) as the data obtained from simple screening tests.

The screening tests we used can only offer a basic assessment of inattention/field-defect present or absent. A potential advantage of the app is that it produces quantitative measures of inattention (laterality index) and fields (construction of a field map that emulates outputs from perimetry). We believe this more nuanced data offer greater clinical information than screening and could, in theory, be used to chart change over time in practice or in the setting of a clinical trial. Other potential pragmatic advantages not captured in the present research activity relate to compatibility with the electronic patient record, and the ability to share results with patients, carers, and extended members of the multidisciplinary team. Designed for use in the NHS setting, reports can be cascaded *via* secure email. While confrontation techniques vary markedly between clinicians, standardization of such facets as screen contrast, target speed, together with automated calculation, and incorporation of reaction time, offer important advantages in terms of standardization and recording of visual field and inattention results. However, such potential advantages would need to be more robustly evaluated in a larger pragmatic trial. Limitations when compared to the gold standard perimetry tests relate to the absence of chin and head rests, a single high contrast isopter being charted, smaller testable field, and less formal fixation monitoring.

As the Stroke Vision app was developed for use in clinical practice, we felt that demonstrating feasibility of testing was an important consideration. Our results show the difficulty of testing in an acute and subacute stroke population. Many participants struggled to complete the various screening, app-based, and gold standard assessments. Our quantitative assessments of user and tester acceptability are reassuring. For our bespoke, simple assessments of user and tester acceptability we did not perform formal comparative analyses. However, the basic descriptive statistics suggest that compliance and acceptability of the app are similar to current screening tools and possibly better than current gold standard assessments. Time required for app assessment is increased compared to usual screening, albeit the total time to complete the suite of app assessments is still relatively short (around 7 min) and would not preclude use of the app in a clinical setting.

The acuity test is the first assessment within StrokeVision, and serves as a prompt to ensure a near spectacle correction that is worn; it also provides a means to gauge the *detection acuity* at a typical reading distance (33 cm). This ensures subsequent assessments for a given eye are up-scaled appropriately, such that low acuity does not preclude assessment of field/inattention. This divergence in design, when compared to conventional standards, represents a source of variation, and a reason results may have been obtained with StrokeVision, but not with conventional bedside/gold standard methods. The strengths of our approach include a “real world” clinical study design that allows description of test properties in the healthcare context in which the app may be used. We used an “intention to diagnose” approach to quantifying (17) our accuracy assessments. This incorporates those instances, where testing was not possible or incomplete and highlights the inherent problems in test accuracy studies that involve participants with physical, visuospatial, and cognitive impairments (18). We also followed best practice in conduct and reporting using guidance for neuropsychological test accuracy studies (19). There are limitations to our approach. Although we based our sample size on a pre-specified power calculation, the wide confidence intervals around our accuracy measure suggests a degree of uncertainty and a study with a greater number of participants would be preferred. Our real world sampling frame gave us a limited number of participants with inattention issues, and so the confidence intervals around these measures are particularly wide. A future test accuracy study may wish to “enrich” the sample with higher number of participants with suspected inattention issues.

Our data suggest that the app is at least as good as traditional bedside screening. One interpretation of these data would be that we should continue the *status quo* of screening using finger-based confrontation. Although not captured by our test metrics, the app offers other functionality that makes it potentially attractive for use in acute stroke settings. The app output offers a map of visual fields that allows a permanent visual record which can be stored and shared. The ability to chart fields at the bedside, rather than the patient having to move to an area with perimetry was appreciated by patients and staff. The app offers more than diagnostic assessment and based on the results of testing, the app can link to information resources for patients and staff. A further feature that was particularly welcomed by patients is the ability of the app to use the assessment information to modify the images produced by the tablet’s camera and create a view of the world as the patient perceives it (e.g., with a field defect, etc).

There are other apps and software that have been developed to assess field deficits, but from a screen of app stores (at the time of writing) there are no commercially available apps with a stroke focus that assess acuity, fields, and inattention. The test properties of our app compare favorably with other apps and software available. A virtual reality test battery for assessing neglect has been developed (11), but is reliant on taking the patient to a computer and requires wearing virtual reality headset for the duration of the test—so while that test battery does address the need for digital assessments, it falls short of providing an easy-to-use bedside tool. A more portable computerized assessment based on the BIT has been described and authors found their digital methods which were more objective and allowed for the collection of what they called “dynamic” data i.e., it was possible to gather information on the starting points and pattern of cancelation in cancelation tasks (12). This may help in identifying subtle neglect and in exploring the heterogeneous nature of the condition (13).

We believe we have demonstrated proof of concept and sufficient accuracy, feasibility, and acceptability to warrant further testing of the utility of StrokeVision. The next phase of testing would take an implementation science approach and address whether staff make use of the app, and whether this leads to improvements in care. This study focused on app-based patient assessments, however, the app offers other potential functionality as an education, training, and information source and these aspects would benefit from further assessment. Field deficits and inattention are only some of the stroke-related visual problems seen in practice. Further development of the app could be major on eye movement disorders using eye tracking software.

In conclusion, we have developed an app for assessment of stroke-related visual problems that has demonstrated acceptable test properties in a stroke population. The app is now ready for larger scale evaluation. We look forward to seeing the app being used in further assessments and welcome feedback from clinicians, patients, and other stake holders.

## Ethics Statement

*Study Design*: We conducted a prospective test accuracy study, recruiting patients from various acute stroke sites across a University Hospital stroke service. The protocol was registered and is available at clinical trials (NCT02539381). We followed Standard for Reporting Diagnostic Accuracy (STARD) best practice in design, conduct, and reporting of our study. Our project was approved by West of Scotland NHS Research Ethics (15/WS/0071) and local Research and Development. All subjects gave written informed consent in accordance with the Declaration of Helsinki. The work was supported by the National Advisory Committee for Stroke (NACS). The funder played no part in data collection, analysis, or interpretation.

## Author Contributions

TQ, IL, AW, CM, and CT helped in design of the study, development of the app, analysis of data, drafting the manuscript, and in critical revision of the manuscript. RS, AB helped in patient recruitment, patient assessment, drafting the manuscript, and critical revision of the manuscript.

## Conflict of Interest Statement

The authors declare that the research was conducted in the absence of any commercial or financial relationships that could be construed as a potential conflict of interest.
